# OcUGT1-Catalyzing Glycodiversification of Steroids through Glucosylation and Transglucosylation Actions

**DOI:** 10.3390/molecules25030475

**Published:** 2020-01-22

**Authors:** Yan-Li Xu, Jian-Qiang Kong

**Affiliations:** Institute of Materia Medica, Chinese Academy of Medical Sciences & Peking Union Medical College (State Key Laboratory of Bioactive Substance and Function of Natural Medicines & NHC Key Laboratory of Biosynthesis of Natural Products), Beijing 100050, China; yanlixu@imm.ac.cn

**Keywords:** glycodiversification, glucosylation, transglucosylation, glycosyltransferase, steroidal glycosides

## Abstract

Steroidal glycosides are important sources of innovative drugs. The increased diversification of steroidal glycosides will expand the probability of discovering active molecules. It is an efficient approach to diversify steroidal glycosides by using steroidal glycosyltransferases. OcUGT1, a uridine diphosphate-d-glucose (UDP-Glc)-dependent glycosyltransferase from *Ornithogalum caudatum*, is a multifunctional enzyme, and its glycodiversification potential towards steroids has never been fully explored. Herein, the glycodiversification capability of OcUGT1 towards 25 steroids through glucosylation and transglucosylation reactions were explored. Firstly, each of 25 compounds was glucosylated with UDP-Glc. Under the action of OcUGT1, five steroids (testosterone, deoxycorticosterone, hydrocortisone, estradiol, and 4-androstenediol) were glucosylated to form corresponding mono-glucosides and biosides. Next, OcUGT1-mediated transglucosylation activity of these compounds with another sugar donor *ortho*-nitrophenyl-β-d-glucopyranoside (*o*NPGlc) was investigated. Results revealed that the same five steroids could be glucosylated to generate mono-glucosides and biosides by OcUGT1 through transglucosylation reactions. These data indicated that OcUGT1-assisted glycodiversification of steroids could be achieved through glucosylation and transglucosylation reactions. These results provide a way to diversify steroidal glycosides, which lays the foundation for the increase of the probability of obtaining active lead compounds.

## 1. Introduction

Steroidal glycosides (SGs) possess broad biological activities, such as anti-inflammatory effect [1,2], anticancer activity [3,4,5], antifungal property [6,7,8], and antiviral activity [9,10]. Moreover, steroidal glycosides are able to serve as the precursors of pharmaceutical steroids [11]. These data indicate that steroidal glycosides are a kind of important natural products with pharmaceutical potential. Hence, it is necessary to diversify steroidal glycosides, so as to increase the probability of discovering innovative drugs from steroidal glycosides. The diversification of SGs, namely glycodiversification of steroids, was achieved by chemical and enzymatical strategies.

Glycodiversification is a synthetic process, through which the structural diversity of carbohydrates is expanded [12]. Owing to the structural complexity of SGs, glycodiversification of steroids by chemical synthesis may be a formidable task [12]. Conversely, the enzymatic glycodiversification is becoming a main strategy for diversifying glycosylated natural products due to the great strides made in the generation of glycosyltransferases (GTs) with catalytic promiscuity [13,14,15]. Glycosyltransferase is the major biocatalyst of enzyme-based glycodiversification, transferring sugar moieties from activated donor molecules (such as nucleotide-sugars) to acceptor substrates with a regio- and stereo-selective manner. The glycodiversification of natural products was thus dependent on the promiscuity of gylcosyltransferase. Although many steroidal glycosyltransferases (SGTs) had been isolated from varied species within the last few years [11,16,17,18,19,20,21,22], most SGTs had not been fully explored for their glycodiversification potentials, including the multifunctional glycosyltransferase OcUGT1 [23]. In our previous reports, OcUGT1 was characterized to be a UDP-Glc-dependent glycosyltransferase with a catalytic flexibility, catalyzing diverse aglycons to form corresponding glycosides [23,24]. Moreover, OcUGT1 was observed to glycosylate testosterone to generate its glycosides, indicating OcUGT1 was able to act as a SGT [25]. Therefore, it is of great significance to carry out the research of OcUGT1 on glycodiversification of steroids for enriching the steroidal glycosides and expanding the enzymatic tools for glycodiversification of steroids.

Herein, the glycodiversification potential of a purified OcUGT1 towards steroids in both glucosylation and transglucosylation reactions was explored. Results indicated that OcUGT1 was able to attack 17β-OH and the primary hydroxyl group at C-21 position of steroids. Moreover, OcUGT1 was demonstrated to have the ability of multiple glycosylations, accepting its glycosylated products for further attachment, no matter the glycosylation and transglycosylation reactions. Cumulatively, OcUGT1 may be deemed as a potential enzymatic tool for glycodiversification of steroids.

## 2. Results and Discussion

### 2.1. Intracellular Expression and Purification of OcUGT1

The recombinant plasmid pET28a-OcUGT1 were introduced into *E. coli* BL21 (DE3) harboring a chaperone plasmid pKJE7 for heterologous expression, respectively [23]. The expression procedure was the same as that previously reported [23,24,25,26]. The expressed OcUGT1 were verified by 12% SDS-PAGE electrophoresis where an intense band with 53 kDa were readily visible (Figure S1), suggesting successful expression of OcUGT1.

OcUGT1 was then purified to near homogeneity using an affinity chromatography as described previously [23,24,25,26]. The purified protein was quantified to be 10.5275 mg/mL. The purified OcUGT1 was then used as a biocatalyst for the glycodiversification of steroids.

### 2.2. OcUGT1-Catalyzed Glucosylation Towards Steroids

Previous studies had shown that OcUGT1 was able to glycosylate testosterone [23]. The glycodiversification capability of OcUGT1 towards steroids, however, had not been documented. Herein, a total of 25 steroids were used as the substrate to react with OcUGT1 to test the glycodiversification potential of OcUGT1. The results showed that five compounds, namely testosterone (**1**), deoxycorticosterone (**2**), hydrocortisone (**3**), estradiol (**4**), and 4-androstenediol (**5**), were able to form new peaks under the catalyzation of OcUGT1.

**Figure 1:**
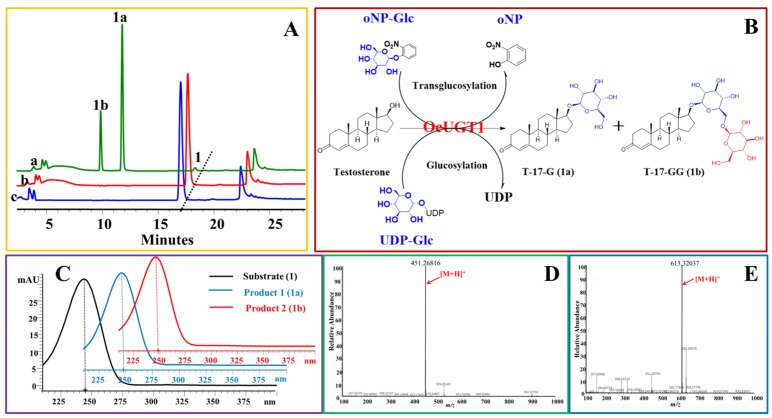
OcUGT1-catalyzed glucosylation of testosterone (**1**). (**A**) HPLC profiles of testosterone (**1**) glucosylation. (**a**) the reaction mixture of testosterone (**1**) with the purified OcUGT1; (**b**) the reaction mixture of testosterone (**1**) without the purified OcUGT1; (**c**) the authentical standard testosterone (**1**); **1**, **1a,** and **1b** refer to testosterone (**1**) and its mono-glucoside and bioside, respectively. (**B**) OcUGT1-mediated glucosylation of testosterone. (**C**) Ultraviolet (UV) spectra of testosterone (**1**) and its glucosides. (**D**) HR-ESI-MS spectrum of T-17-G (**1a**). (**E**) HR-ESI-MS spectrum of T-17-GG (**1b**).

Two new peaks, **1a** and **1b**, appeared after the incubation of testosterone (**1**) with purified OcUGT1 at 50 °C for 2.5 h (Figure 1A), which was consistent with the previous report [25]. Both **1a** and **1b** displayed similar UV spectra with that of testosterone (**1**), indicating three compounds shared a similar backbone structure (Figure 1C). Metabolites **1a** and **1b** were found with *m/z* of 451.26816 [M + H]^+^ and 613.32037 [M + H]^+^, corresponding to their mono-glucosylation (C_25_H_38_O_7_) and di-glucosylation (C_31_H_48_O_12_) of testosterone, respectively (Figure 1D–E). Herein, di-glucosylation refers to testosterone, in principle, that could be glycosylated at two different positions (di-glucoside) or the glycosyl moiety in testosterone mono-glucoside could react with a second molecule of glucose (bioside). Further, 1D-NMR (^1^H- and ^13^C-NMR) data supported that **1a** and **1b** were monoside and bioside of testosterone (Table 1 and Table 2, Figures S2–15), respectively. The locations of glucose group in **1a** and **1b** were determined based on their HMBC data (Figure S5,12). The HMBC correlation at δH/δC 4.32 (H-1′)/89.6(C-17) demonstrated that Glc 1 was located at C-17 of the testosterone aglycone, which was consistent with our previous study [25]. Another HMBC correlation at δH/δC 4.35 (H-1″)/69.7(C-6ʹ) confirmed that Glc 2 was located at C-6 of Glc 1. Correlations also observed in the HMBC spectrum of **1b** between the H-6ʹa of Glc 1 at δH 4.12 and C-1 of Glc 2(C-1″) at δc 104.8, H-6ʹb of Glc 1 at δH 3.79, and C-1 of Glc 2(C-1″) at δc 104.8 established Glc 2 was located at C-6ʹ –OH of the mono-glucoside. These data collectively assigned **1a** and **1b** to be testosterone 17-*O*-β-d-glucoside (T-17-G) (Table 1, Figures S2–S8) and testosterone 17-*O*-β-d-glucopyranosyl-(1→6)- β-d-glucopyranoside (T-17-GG) (Table 2, Figures S9–S15). Previously, only the mono-glucoside of testosterone catalyzed by OcUGT1 had been identified exactly [25]. Herein, besides the mono-glycoside, the structure of testosterone bioside has been identified to testosterone 17-*O*-β-d-glucopyranosyl-(1→6)- β-d-glucopyranoside (testosterone 17-*O*-gentobioside), which is a new compound. These results collectively indicated that OcUGT1 was able to exert glucosylation on the 17β-position of testosterone (Figure 1B). When epitestosterone (**7**), an epimer of testosterone (**1**), was incubated with OcUGT1, no new peaks were present in the reaction mixture, suggesting OcUGT1 had no activity towards the hydroxyl group on 17α position of epitestosterone (**7**). This notion was further confirmed that OcUGT1 had no effect on 17α-OH of 17α-hydroxypregnenolone (**11**) and 17α-hydroxyprogesterone (**15**). These data indicated that the OcUGT1-catalyzed glucosylation is a stereospecific reaction, in which the hydroxyl groups with β-configuration can be specifically glucosylated. However, OcUGT1-mediated glucosylation towards 17β-OH could be hindered by the introduction of an ethynyl group. Therefore, no products were yielded in OcUGT1-assisted glucosylation towards ethisterone (**14**).

**Figure 2:**
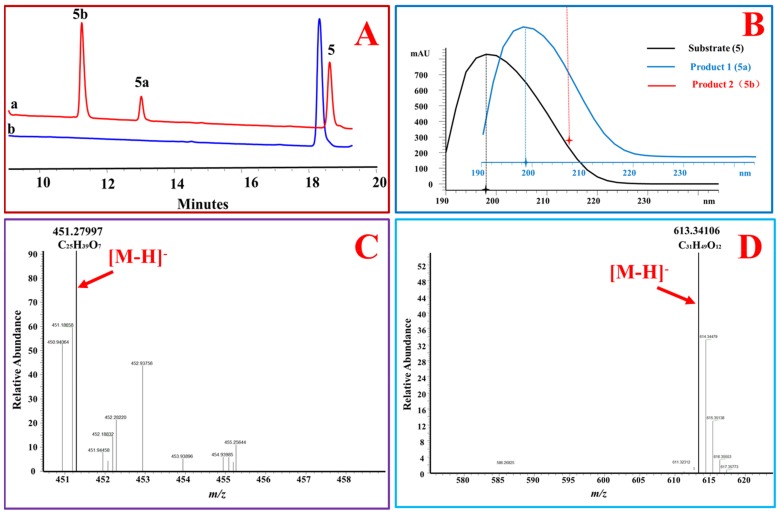
OcUGT1-catalyzed glucosylation of 4-androstenediol (**5**). (**A**) HPLC profiles of 4-androstenediol (**5**) glucosylation. (**a**) the reaction mixture of 4-androstenediol (**5**) with the purified OcUGT1; (**b**) the reaction mixture of 4-androstenediol (**5**) without the purified OcUGT1; **5**, **5a,** and **5b** refer to 4-androstenediol (**5**) and its mono-glucoside and bioside, respectively. (**B**) UV spectra of 4-androstenediol (**5**) and its glucosides. (**C**) HR-ESI-MS spectrum of 4-androstenediol mono-glucoside (**5a**). (**D**) HR-ESI-MS spectrum of 4-androstenediol bioside (**5b**).

Moreover, the position of a double bond in steroidal skeleton affected the glycosylation activity of OcUGT1. OcUGT1 was able to glucosylate 17β-OH when a double bond was at the Δ4 position of a steroid (4-androstenediol (**5**) and testosterone (**1**)) (Figure 1 and Figure 2). OcUGT1-directed glucosylation towards 17β-OH did not occur if a double bond was at the Δ5 position (5-androstenediol (**6**)).

**Figure 3:**
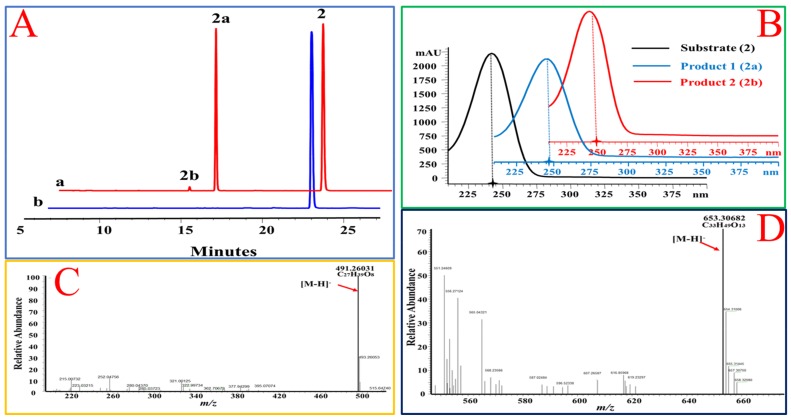
OcUGT1-catalyzed glucosylation of deoxycorticosterone (**2**). (**A**) HPLC profiles of deoxycorticosterone (**2**) glucosylation. (**a**) The reaction mixture of deoxycorticosterone (**2**) with the purified OcUGT1; (**b**) the reaction mixture of deoxycorticosterone (**2**) without the purified OcUGT1; **2**, **2a,** and **2b** refer to deoxycorticosterone (**2**) and its mono-glucoside and bioside, respectively. (**B**) UV spectra of deoxycorticosterone (**2**) and its glucosides. (**C**) HR-ESI-MS spectrum of deoxycorticosterone mono-glucoside (**2a**). (**D**) HR-ESI-MS spectrum of deoxycorticosterone bioside (**2b**).

**Figure 4:**
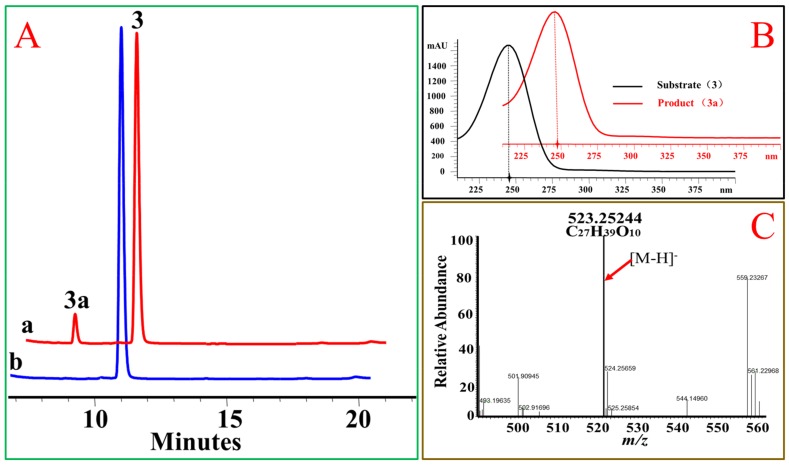
OcUGT1-catalyzed glucosylation towards hydrocortisone (**3**). (**A**) HPLC profiles of hydrocortisone (**3**) glucosylation. (**a**) the reaction mixture of hydrocortisone (**3**) with the purified OcUGT1; (**b**) the reaction mixture of hydrocortisone (**3**) without the purified OcUGT1. **3** and **3a** refer to hydrocortisone (**3**) and its mono-glucoside, respectively. (**B**) UV spectra of hydrocortisone (**3**) and its glucosides. (**C**) HR-ESI-MS spectrum of hydrocortisone mono-glucoside (**3a**).

In addition, the 17β hydroxyl group, OcUGT1 was able to transfer glucose to primary hydroxyl group at C-21 position in the side chain of steroids. When deoxycorticosterone (**2**) was incubated with OcUGT1, two new peaks with similar UV spectra to that of the substrate were present in the mixture (Figure 3). The molecular formula of the major product (**2a**) was established as C_27_H_39_O_8_ by HRESIMS at *m/z* 491.26031 [M − H]^−^, corresponding to the mono-glucoside of deoxycorticosterone (**2**). The minor product (**2b**) gave a molecular formula of C_33_H_49_O_13_ by HRESIMS at *m/z* 653.30682 [M − H]^−^, corresponding to the di-glucosylation of deoxycorticosterone (**2**). Based on the catalytic behavior of OcUGT1 and the fact that deoxycorticosterone contained only one hydroxyl group at C-21 position, the mono-glucoside was deduced to be deoxycorticosterone 21-d-glucoside (Figure 3). Moreover, a mono-glucosylated product was yielded in OcUGT1-guided glucosylation towards hydrocortisone (**3**) (Figure 4). Both deoxycorticosterone (**2**) and hydrocortisone (**3**) have a primary hydroxyl group at C-21 position. The mono-glucosylated hydrocortisone was thus deduced to be hydrocortisone 21-d-glucoside (Figure 4). However, OcUGT1 did not attach the glucosyl group to a secondary hydroxyl group in the side-chain of steroids (cyasterone (**19**) and 24-epicastasterone (**16**)).

**Figure 5:**
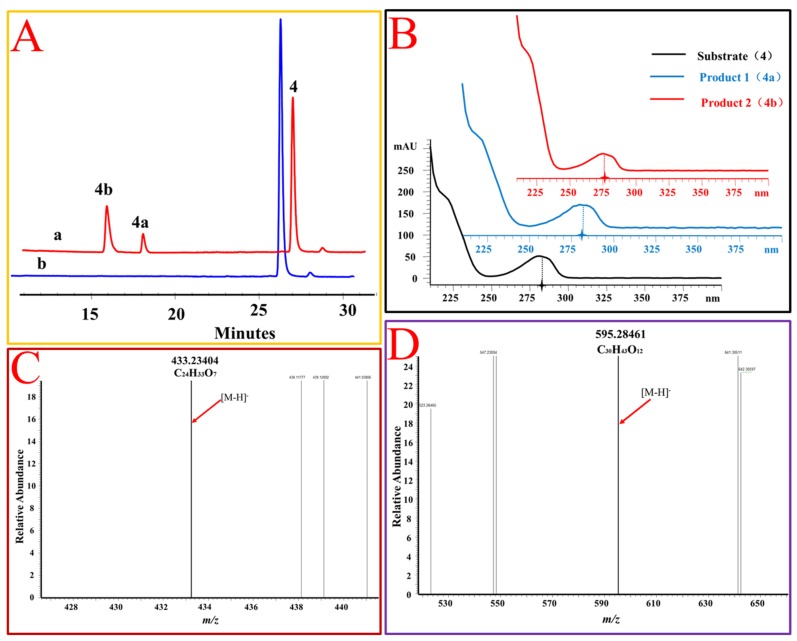
OcUGT1-catalyzed glucosylation of estradiol (**4**). (**A**) HPLC profiles of estradiol (**4**) glucosylation. (**a**) the reaction mixture of estradiol (**4**) with the purified OcUGT1; (**b**) the reaction mixture of estradiol (**4**) without the purified OcUGT1. **4**, **4a,** and **4b** refer to estradiol (**4**) and its mono-glucoside and bioside, respectively. (**B**) UV spectra of estradiol (**4**) and its glucosides. (**C**) HR-ESI-MS spectrum of estradiol mono-glucoside (**4a**). (**D**) HR-ESI MS spectrum of estradiol bioside (**4b**).

**Figure 6:**
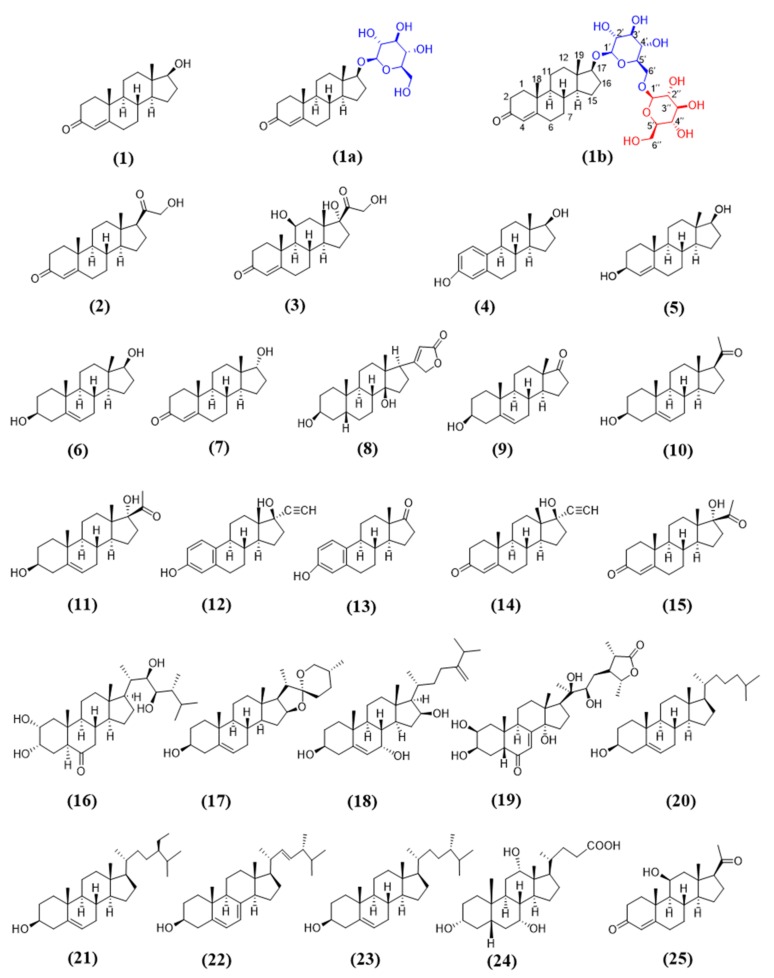
Steroidal substrates used in this study.

The hydroxyl group at C-3 position of steroids is the site attacked by most known SGTs [11,18]. However, OcUGT1 displayed no activity towards the hydroxyl group at C-3 position, which was evidenced by the fact that OcUGT1 did not react with steroids with C3-OH, such as cerberigenin (**8**), dehydroepiandrosterone (**9**), pregnenolone (**10**), 17α-hydroxypregnenolone (**11**), diosgenin (**17**), cholesterol (**20**), β-sitosterol (**21**), ergosterol (**22**), campesterol (**23**), and cholic acid (**24**). In addition to the alcoholic hydroxyl group on C-3 position, OcUGT1 cannot glucosylate the phenol hydroxyl group at C-3 position of ethinyl estradiol (**12**) and estrone (**13**). Moreover, OcUGT1 was determined to display no glucosylation activity towards hydroxyl groups at C-2(24-epicastasterone (**16**) and cyasterone (**19**)), C-7(ergosta-5,24(28)-diene-3,7,16-triol (**18**) and cholic acid (**24**)), C-11(11β-hydroxyprogesterone (**25**)), C-12 (cholic acid (**24**)), C-14 (cerberigenin (**8**) and cyasterone (**19**)), or C-16 (ergosta-5,24(28)-diene-3,7,16-triol (**18**)) position of steroids. Therefore, the glucosylated product of estradiol (**4**) with a free phenolic hydroxyl group at C-3 position catalyzed by OcUGT1 was reasonably deduced as estradiol 17-*O*-β-d-glucoside (**4a**) and corresponding bioside (**4b**) based on the MS data and the catalytic behavior of OcUGT1 (Figure 5). Cumulatively, OcUGT1 was able to attack 17β-OH and the primary hydroxyl group at C-21 position of steroids (Table 3 and Figure 6).

### 2.3. OcUGT1-Mediated Transglucosylation Towards Steroids

**Figure 7:**
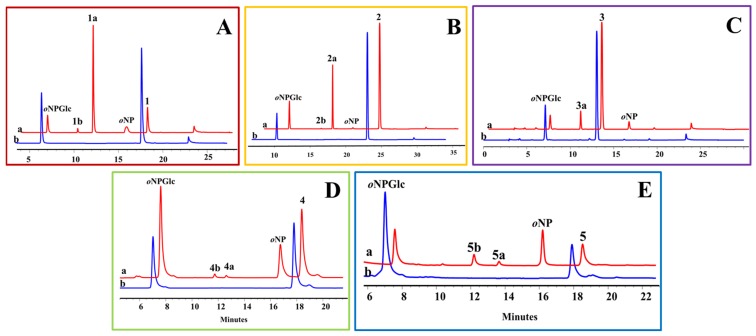
HPLC profiles of OcUGT1-assisted transglucosylation between *o*NPGlc and testosterone (**A**), deoxycorticosterone (**B**), hydrocortisone (**C**), estradiol (**D**) or 4-androstenediol (**E**). (**a**) Transglucosylation reaction with purified OcUGT1; (**b**) transglucosylation reaction without purified OcUGT1.

In OcUGT1-directed glucosylations towards steroids, an expensive compound UDP-Glc (2450 ¥/g, J&K Scientific Ltd.) was used as the sugar donor, which was not conducive to the diversification of steroidal glycosides. Therefore, OcUGT1-mediated transglycosylations using a cheaper aryl-substituted glycoside *o*NPGlc (770 ¥/g, J&K Scientific Ltd.) as a sugar donor were tested in this investigation. Under the action of OcUGT1, each of the 25 compounds reacted with *o*NPGlc separately. When testosterone (**1**) was incubated with *o*NPGlc, OcUGT1 catalyzed the sugar transfer from *o*NPGlc to testosterone, forming testosterone mono-glucoside T-17-G (**1a**). Furthermore, OcUGT1 transferred the sugar group from *o*NPGlc to the glucosyl moiety of T-17-G (**1a**) to form the corresponding testosterone bioside T-17-GG (**1b**). Meanwhile, *o*NPGlc was deglucosylated to form *o*NP (Figure 7A). These data indicated that OcUGT1 has the ability of multiple glucosylations in the process of transglucosylation, which was the same as that of glucosylation reactions. This notion was further verified by the transglucosylation reactions between *o*NPGlc and deoxycorticosterone (**2**) (Figure 7B), estradiol (**4**) (Figure 7D) or 4-androstenediol (**5**) (Figure 7E), in which steroidal mono-glucosides and biosides were generated. Moreover, OcUGT1-assisted transglycosylation towards hydrocortisone (**3**) resulted in only one mono-glucoside, consistent with that of OcUGT1-catalyzed hydrocortisone (**3**) glucosylation (Figure 7C). With the exception of the above five steroids, the remaining 20 compounds had no reactivity with *o*NPGlc. Thus, the substrate spectrum of OcUGT1-assisted transglucosylation is consistent with that of glucosylation reactions. In addition, the product diversity generated through transglucosylations is the same as that of glucosylations. These data collectively revealed that both transglucosylation and glucosylation can achieve a considerable glycodiversification of steroids.

## 3. Materials and Methods

### 3.1. Plasmids and Strains

The expression plasmid pET28a-OcUGT1, constructed in our previous report [23], was used for heterologous expression of the *OcUGT1* gene. The *Escherichia coli* strains *Tran*s1-T1 and BL21 (DE3) (TransGen Biotech, Beijing, China) were used as the hosts for plasmids amplification and heterologous expression, respectively.

### 3.2. Chemicals and Reagents

Steroidal substrates summarized in Figure 6 were purchased from BioBioPha Co. Ltd. (Kunming, China), Push Bio-technology Co. Ltd. (Chengdu, China), and J&K Scientific Ltd. (Beijing, China), respectively. These steroids were dissolved in dimethyl sulfoxide (DMSO) for glucosylation and transglucosylation assays unless otherwise noted.

### 3.3. Intracellular Expression and Purification of OcUGT1

The plasmid pET28a-OcUGT1 was introduced into a BL21 (DE3) harboring a chaperone plasmid pKJE7 for intracellular expression of OcUGT1 as described previously [23]. The resultant OcUGT1 was then purified to near homogeneity [23]. After being quantified by the Bradford method [27], the purified OcUGT1 was used as the biocatalyst for glucosylation and transglucosylation assays of steroids.

### 3.4. Assays for Glucosylation Activity

The purified OcUGT1-catalyzed glucosylation reaction were carried out as previously described [23], with a minor modification. Briefly, OcUGT1-catalyzed glucosylation assays were run in a 100 μL reaction containing a 10 μL PBS buffer (0.2 M, pH8.0), 10 μL UDPG (10 mM), 10 μL steroid substrate (10 mM), and 10 μL purified OcUGT1. After being run at 50 °C for 2.5 h, glucosylation reactions were terminated by the addition of 100 μL methanol and 10 μL glacial acetic acid. The reaction mixture was then centrifuged at 12,000× *g* for 5 min and the resultant supernatant was filtered through a 0.22 μm filter. The filtered supernatant was directly analyzed by high-performance liquid chromatography (HPLC) as described previously [25].

### 3.5. Assays for OcUGT1-Catalyzed Transglucosylation Action

The procedure of OcUGT1-assisted transglucosylation actions was the same as described previously [23], with a minor modification. Transglucosylation reactions were performed in a total volume of 100 μL harboring 10 μL PBS buffer (0.2 M, pH6.0), 10 μL *o*NPGlc (*ortho*-nitrophenyl-β-d-glucopyranoside, 10 mM), 10 μL steroid substrate (10 mM), and 10 μL purified OcUGT1. Transglucosylation reactions lasted overnight at 37 °C. The reaction termination and HPLC measurement of the reaction mixtures were the same as those of glucosylation reactions.

### 3.6. Analyses and Structural Identification of Steroidal Glucosides

The analyses and structural identification of metabolites were performed by the combinational use of HPLC, high resolution electrospray ionization mass spectroscopy (HR-ESI-MS), and nuclear magnetic resonance (NMR), as described previously [3,11,23,25,28]. Briefly, analytical HPLC was performed in an Agilent HPLC 1200 system (Agilent, Waldbronn, Germany) equipped with a SilGreen C18 column (250 × 4.6 mm id, 5 μm particle size). The mobile phase was composed of 0.1% trifluoroacetic acid in H_2_O (solvent A) and acetonitrile (solvent B). The gradient elution was as follows: 0–5 min, 15% B; 5–20 min, 50% B; 20–28 min, 100% B. The flow rate was 1.0 mL/min. The injection volume was 50 μL and the effluents were monitored at 25 °C by a DAD detector at 243 nm. Glucosylated products were collected on an SEP LC-52 system (SEP. Co. Ltd., Beijing, China) with a YMC C18 preparative column (250 × 10.0 μm ID, 5 μm; YMC Co. Ltd., Kyoto, Japan). The data collection for HR-ESI-MS were carried out on a Thermo Scientific Exactive Orbitrap LC-Mass spectrometer (Thermo Scientific, Waltham, MA, USA). ^1^H-NMR (600 MHz), ^13^C-NMR (151 MHz), and 2D-NMR spectrometric data were recorded with AVANCE III HD 600 NMR spectrometer (Bruker, Rheinstetten, Germany). Chemical shifts are given in δ (ppm) with the solvent (CD_3_OD-*d*4) peaks as references.

## Figures and Tables

**Table 1:** NMR spectroscopic data for compound **1a** (T-17-G), in CD_3_OD (δ in ppm, *J* in Hz).

Compound 1a		
Position	δ_C_	δ_H_
1	36.7	1.72 (1H, ddd, *J* = 14.4, 12.6, 4.2 Hz, H-1α)
		2.10 (1H, ddd, *J* = 12.6, 4.8, 3.0 Hz, H-1β)
2	34.7	2.27(1H, ddd, *J* = 16.2, 4.2, 3.0 Hz, H-2α)
		2.46 (1H, ddd, *J* = 16.2, 14.4, 4.8 Hz, H-2β)
3	202.3	-
4	124.1	5.70 (1H, s, H-4)
5	175.2	-
6	33.9	2.32 (1H, ddd, *J* = 14.4, 4.2, 2.4 Hz, H-6α)
		2.50 (1H, ddd, *J* = 14.4, 13.8, 5.4 Hz, H-6β)
7	32.8	1.04 (1H, dddd, *J* = 13.8, 12.6, 11.4, 4.2 Hz, H-7α)
		2.02 (1H, dddd, *J* = 12.6, 5.4, 3.0, 2.4 Hz, H-7β)
8	36.8	1.68 (1H, dddd, *J* = 11.4, 10.8, 10.2, 3.0 Hz, H-8)
9	55.5	0.97 (1H, ddd, *J* = 12.0, 10.2, 4.2 Hz, H-9)
10	40.0	-
11	21.8	1.62 (1H, dddd, *J* = 13.2, 4.2, 4.2, 3.0 Hz, H-11α)
		1.50 (1H, dddd, *J* = 13.8, 13.2, 12.0, 4.2 Hz, H-11β)
12	38.5	1.21 (1H, ddd, *J* = 13.8, 12.3, 4.2 Hz, H-12α)
		1.89 (1H, ddd, *J* = 12.3, 4.2, 3.0 Hz, H-12β)
13	44.2	-
14	51.7	1.01 (1H, ddd, *J* = 12.3, 10.8, 7.2 Hz, H-14)
15	24.2	1.64 (1H, dddd, *J* = 12.6, 9.6, 7.2, 3.6 Hz, H-15α)
		1.32 (1H, dddd, *J* = 12.6, 12.3, 12.0, 6.0 Hz, H-15β)
16	29.8	1.60 (1H, dddd, *J* = 13.8, 12.0, 8.4, 3.6 Hz, H-16α)
		2.06 (1H, dddd, *J* = 13.8, 9.6, 9.0, 6.0 Hz, H-16β)
17	89.6	3.76 (1H, dd, *J* = 9.0, 8.4 Hz, H-17)
18	12.0	0.90 (3H, s, H-18)
19	17.7	1.24 (3H, s, H-19)
1ʹ	104.7	4.32 (1H, d, *J* = 7.8 Hz, H-1ʹ)
2ʹ	75.4	3.15 (1H, dd, *J* = 9.0, 7.8 Hz, H-2ʹ)
3ʹ	77.9	3.21 (1H, t, *J* = 9.0 Hz, H-3ʹ)
4ʹ	71.7	3.27 (1H, dd, *J* = 9.0, 8.4 Hz, H-4ʹ)
5ʹ	78.2	3.33 (1H, dd, *J* = 8.4, 5.4 Hz, H-5ʹ)
6ʹ	62.8	3.86 (1H, dd, *J* = 12.0, 2.4 Hz, H-6ʹa)
		3.65 (1H, dd, *J* = 12.0, 5.4 Hz, H-6ʹb)

**Table 2:** NMR spectroscopic data for compound **1b** (T-17-GG) in CD_3_OD (δ in ppm, *J* in Hz).

Compound 1b		
Position	δ_C_	δ_H_
1	36.8	1.75 (1H, ddd, *J* = 14.4, 12.6, 4.2 Hz, H-1α)
		2.12 (1H, ddd, *J* = 12.6, 4.8, 3.0 Hz, H-1β)
2	34.8	2.27 (1H, ddd, *J* = 16.2, 4.2, 3.0 Hz, H-2α)
		2.50 (1H, ddd, *J* = 16.2, 14.4, 4.8 Hz, H-2β)
3	202.4	-
4	124.1	5.70 (1H, s, H-4)
5	175.3	-
6	33.9	2.33 (1H, ddd, *J* = 14.2, 4.2, 2.4 Hz, H-6α)
		2.50 (1H, ddd, *J* = 14.2, 13.8, 5.4 Hz, H-6β)
7	32.8	1.08 (1H, dddd, *J* = 13.8, 12.6, 11.4, 4.2 Hz, H-7α)
		2.04 (1H, dddd, *J* = 12.6, 5.4, 3.0, 2.4 Hz, H-7β)
8	36.8	1.70 (1H, dddd, *J* = 11.4, 10.8, 10.2, 3.0 Hz, H-8)
9	55.4	0.99 (1H, ddd, *J* = 12.0, 10.2, 4.2 Hz, H-9)
10	40.0	-
11	21.8	1.64 (1H, dddd, *J* = 13.2, 4.2, 4.2, 3.0 Hz, H-11α)
		1.53 (1H, dddd, *J* = 13.8, 13.2, 12.0, 4.2 Hz, H-11β)
12	38.3	1.23 (1H, ddd, *J* = 13.8, 12.3, 4.2 Hz, H-12α)
		1.91 (1H, ddd, *J* = 12.3, 4.2, 3.0 Hz, H-12β)
13	44.2	-
14	51.6	1.04 (1H, ddd, *J* = 12.3, 10.8, 7.2 Hz, H-14)
15	24.2	1.67 (1H, dddd, *J* = 12.6, 9.6, 7.2, 3.6 Hz, H-15α)
		1.34 (1H, dddd, *J* = 12.6, 12.3, 12.0, 6.0 Hz, H-15β)
16	29.9	1.61 (1H, dddd, *J* = 13.8, 12.0, 8.4, 3.6 Hz, H-16α)
		2.09 (1H, dddd, *J* = 13.8, 9.6, 9.0, 6.0 Hz, H-16β)
17	89.7	3.79 (1H, dd, *J* = 9.0, 8.4 Hz, H-17)
18	12.0	0.90 (3H, s, H-18)
19	17.7	1.24 (3H, s, H-19)
1ʹ	104.8	4.44 (1H, d, *J* = 7.8 Hz, H-1ʹ)
2ʹ	75.4	3.21 (1H, dd, *J* =9.0, 7.8 Hz H-2ʹ)
3ʹ	77.1	3.29 (1H, t, *J* = 9.0 Hz, H-3ʹ)
4ʹ	71.6	3.36 (1H, dd, *J* = 9.0, 8.4 Hz, H-4ʹ)
5ʹ	78.0	3.37 (1H, dd, *J* = 8.4, 5.4 Hz, H-5ʹ)
6ʹ	69.7	4.12 (1H, dd, *J* = 12.0, 2.4 Hz, H-6ʹa)
		3.79 (1H, dd, *J* = 12.0, 5.4 Hz, H-6ʹb)
1″	104.8	4.35 (1H, d, *J* = 7.8 Hz, H-1″)
2″	75.1	3.21 (1H, dd, *J* =9.0, 7.8 Hz, H-2″)
3″	77.1	3.29 (1H, t, *J* = 9.0 Hz, H-3″)
4″	71.5	3.41 (1H, dd, *J* = 9.0, 8.4 Hz, H- 4″)
5″	78.0	3.37 (1H, dd, *J* = 8.4, 5.4 Hz, H-5″)
6″	62.8	3.67 (1H, dd, *J* = 12.0, 2.4 Hz, H-6″a)
		3.88 (1H, dd, *J* = 12.0, 5.4 Hz, H-6″b)

**Table 3:** Steroids used for OcUGT1-catalyzed glucosylations.

No	Steroid	Reactivity
**1**	testosterone	+
**2**	deoxycorticosterone	+
**3**	hydrocortisone	+
**4**	estradiol	+
**5**	4-androstenediol	+
**6**	5-androstenediol	-
**7**	epitestosterone	-
**8**	cerberigenin	-
**9**	dehydroepiandrosterone	-
**10**	pregnenolone	-
**11**	17α-hydroxypregnenolone	-
**12**	ethinyl estradiol	-
**13**	estrone	-
**14**	ethisterone	-
**15**	17α-hydroxyprogesterone	-
**16**	24-epicastasterone	-
**17**	diosgenin	-
**18**	ergosta-5,24(28)-diene-3,7,16-triol	-
**19**	cyasterone	-
**20**	cholesterol	-
**21**	β-sitosterol	-
**22**	ergosterol	-
**23**	campesterol	-
**24**	cholic acid	-
**25**	11β-hydroxyprogesterone	-

“+” and “-” indicate reactivity and no reactivity with OcUGT1.

## Abbreviations

DMSO:	Dimethyl sulfoxide
GTs:	Glycosyltransferases
HPLC:	High-performance liquid chromatography
NMR:	Nuclear magnetic resonance
*o*NPGlc:	*o**ortho*-nitrophenyl-β-d-glucopyranoside
SGs:	Steroidal glycosides
SGTs:	Steroidal glycosyltransferases
T-17-G:	Testosterone 17-*O*-β-glucoside
T-17-GG:	Testosterone 17-*O*-β-glucopyranosyl-(1→6)-β-d-glucopyranoside
UDP-Glc:	Uridine diphosphate-d-glucose

## Supplementary Materials

The following are available online.
