# Supplementary material for: OcUGT1-Catalyzing Glycodiversification of Steroids through Glucosylation and Transglucosylation Actions
Source: Molecules. 2020 Jan 22;25(3):475. doi: 10.3390/molecules25030475 (PMC7036888; doi:10.3390/molecules25030475)
Supplement: Supplementary file 1 [file molecules-25-00475-s001.pdf]

Supplementary Materials

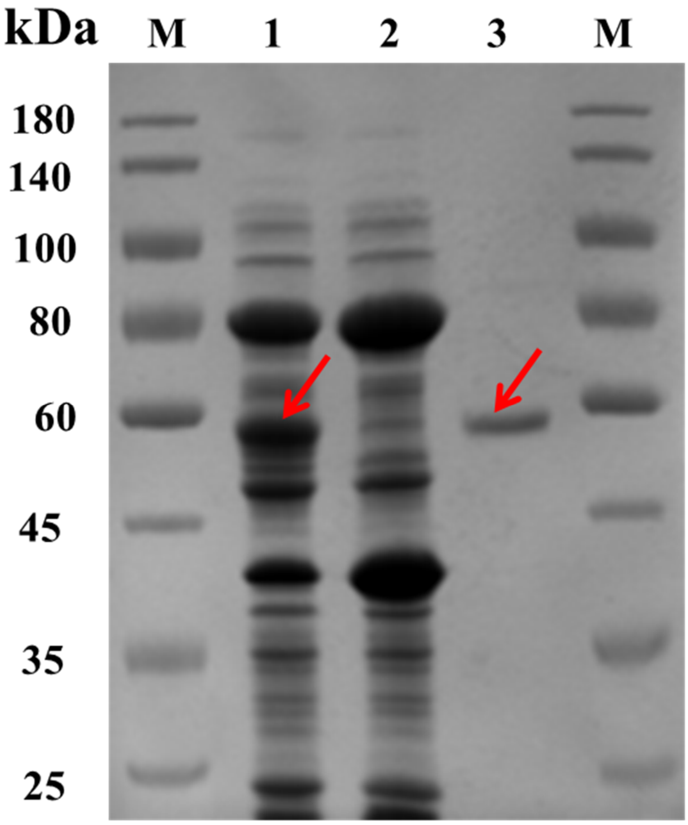

Figure S1. SDS-PAGE analyses of the recombinant OcUGT1 protein. 1, the crude extract of BL21(DE3)[pET28a-OcUGT1+pKJE7]; 2, the crude extract of BL21(DE3)[pET28a +pKJE7]; 3, the purified OcUGT1; M, protein molecular markers; the arrows show the expressed OcUGT1.

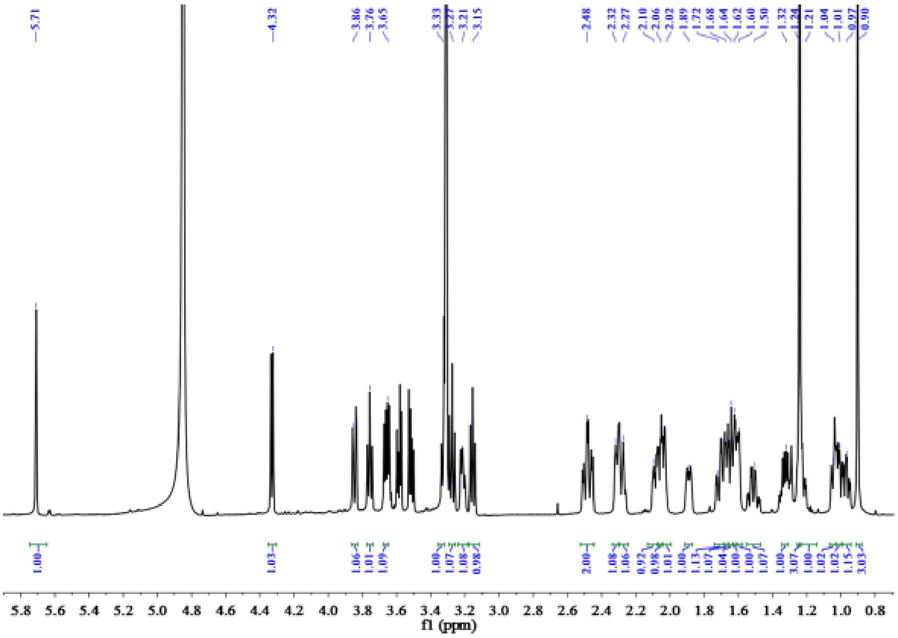

Figure S2. ^{1}H NMR spectrum of **1a** (600 MHz, Methanol-d_4).

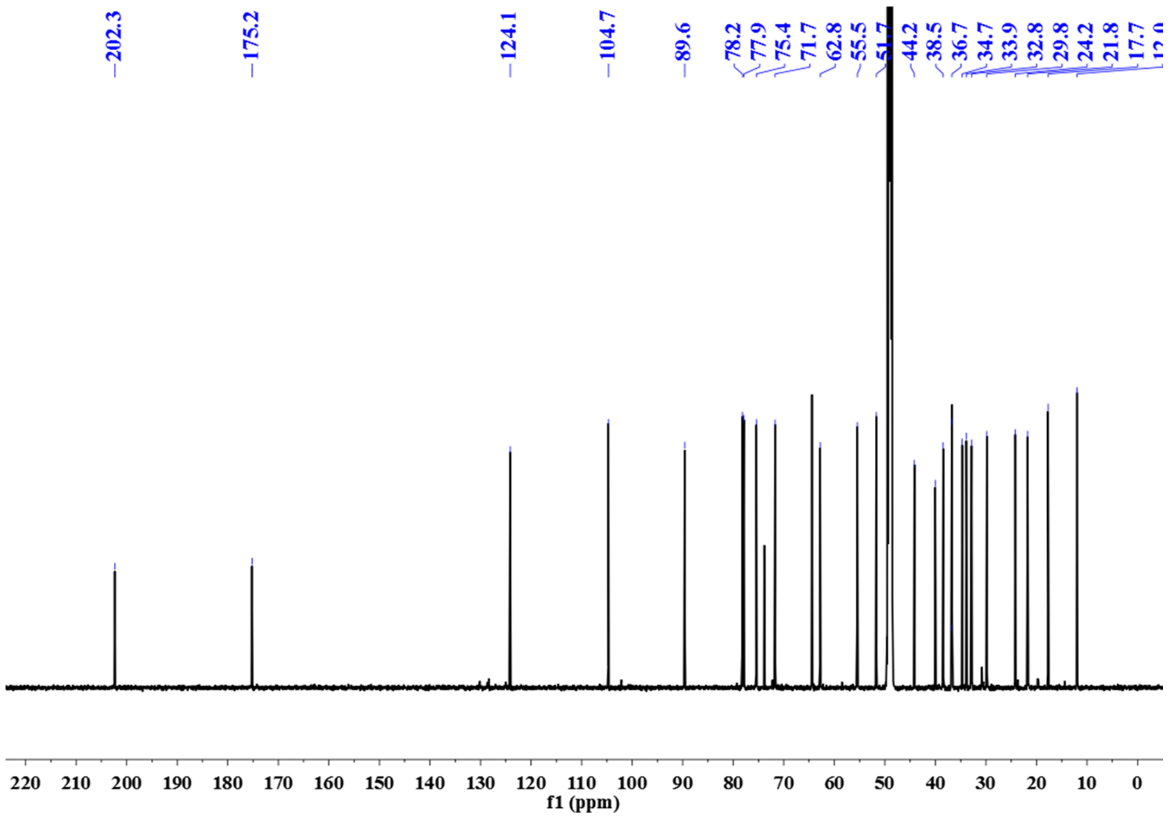

Figure S3. ^{13}C NMR spectrum of **1a** (150 MHz, Methanol-d_4).

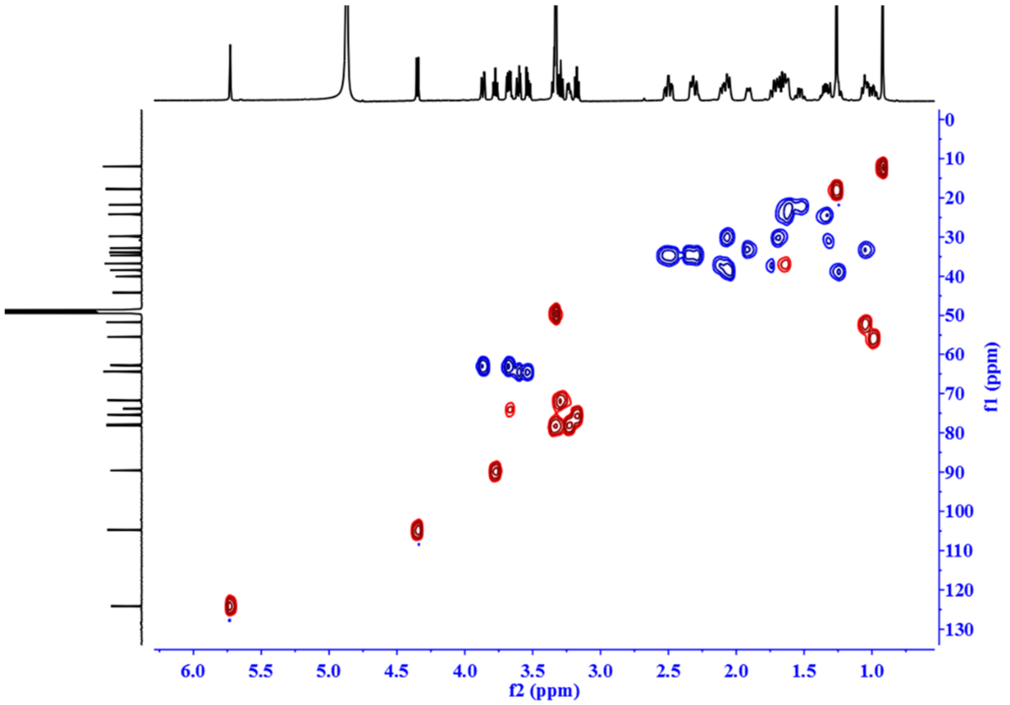

Figure S4. HSQC spectrum of **1a.**

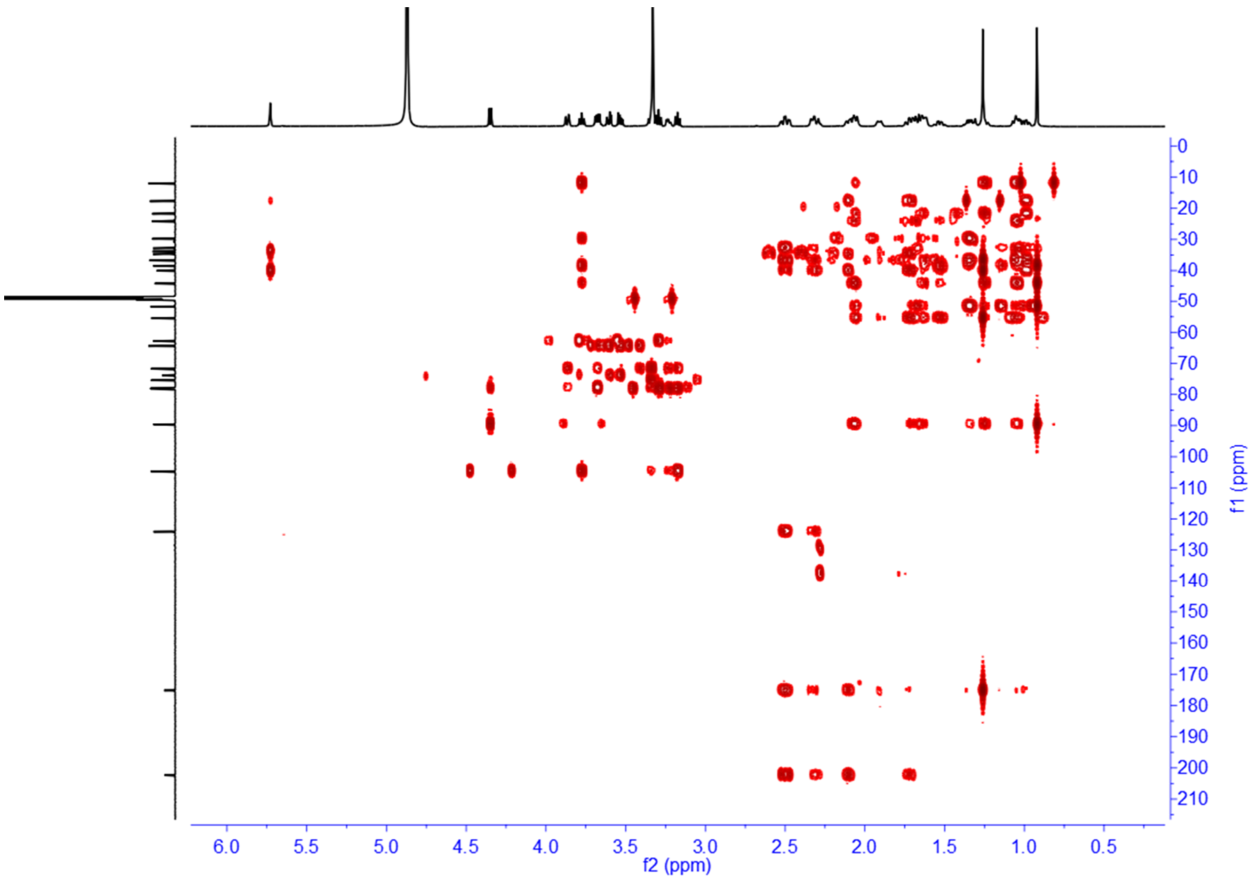

Figure S5. HMBC spectrum of **1a.**

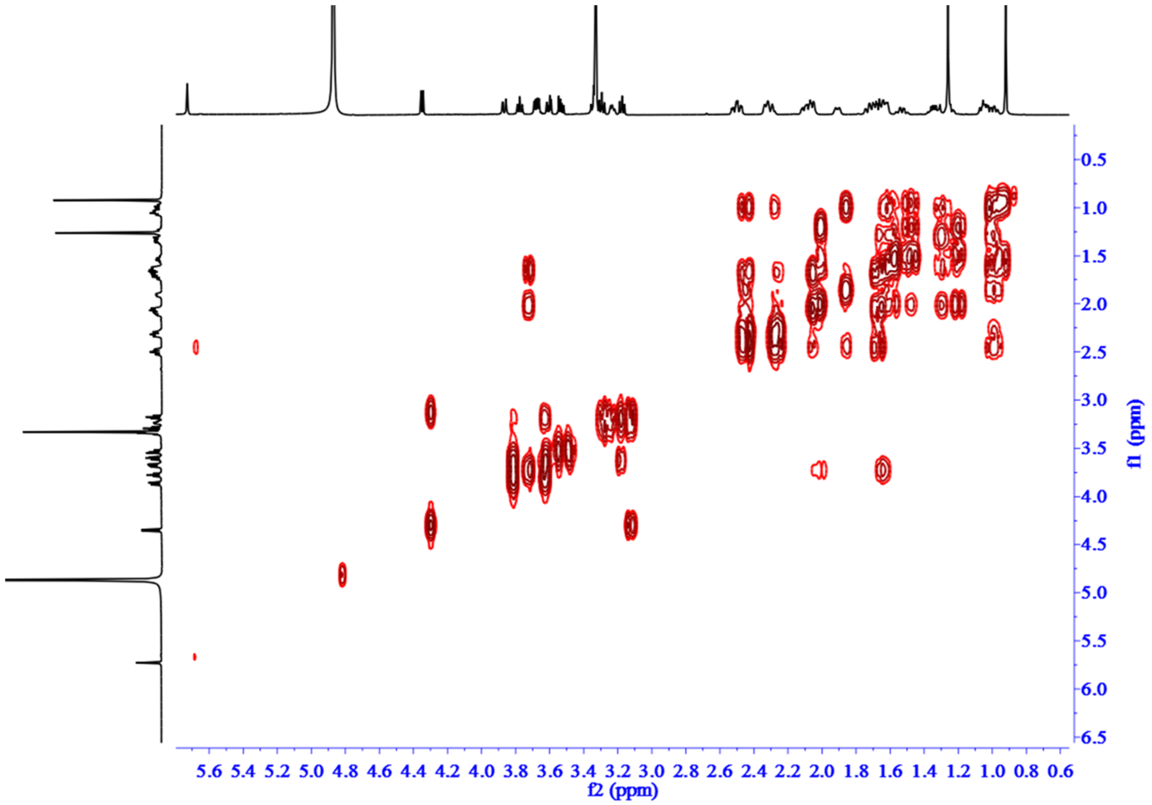

Figure S6. COSY spectrum of **1a.**

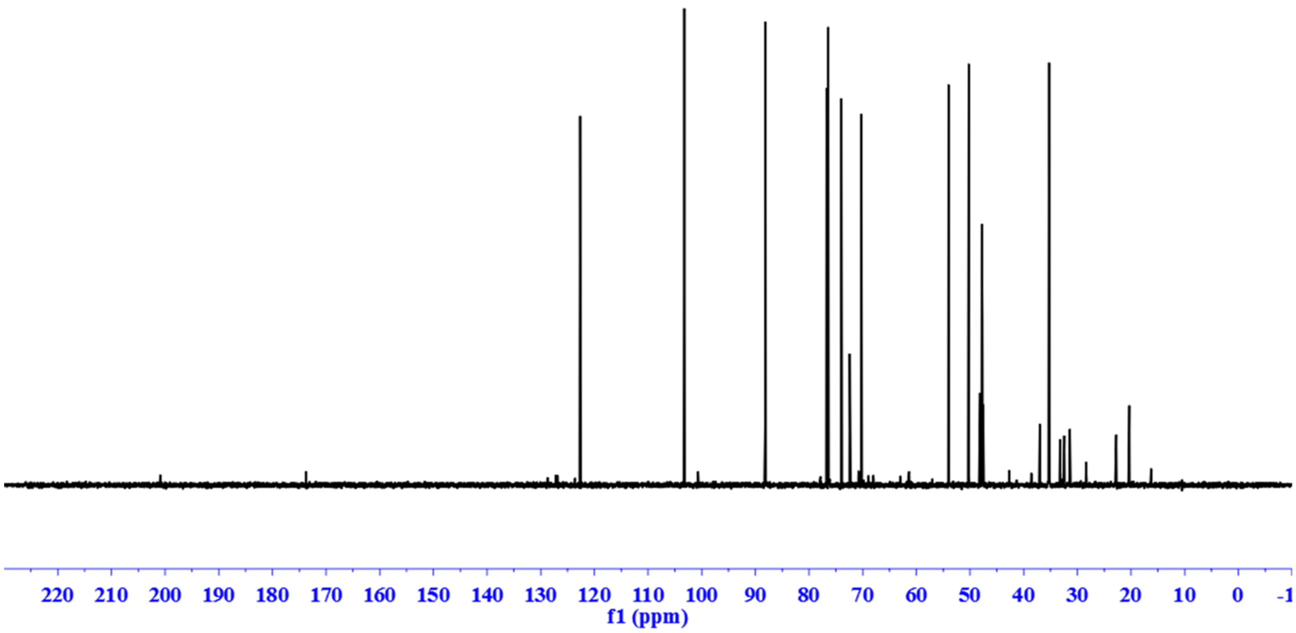

Figure S7. The DEPT-90 ^{13}C spectrum of **1a.**

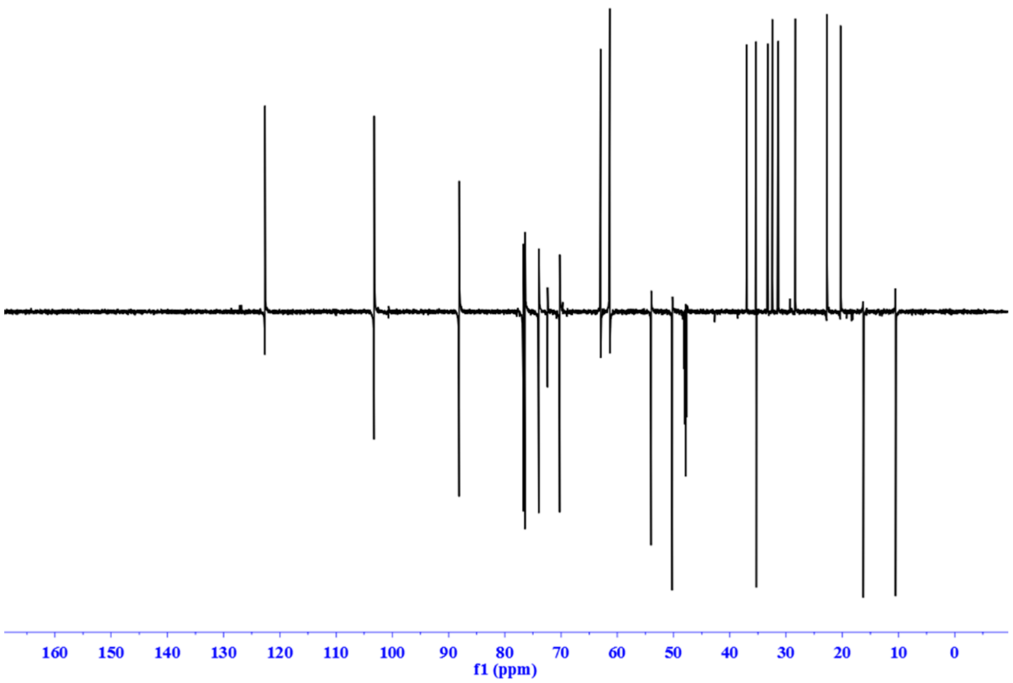

Figure S8. The DEPT-135 ^{13}C spectrum of **1a.**

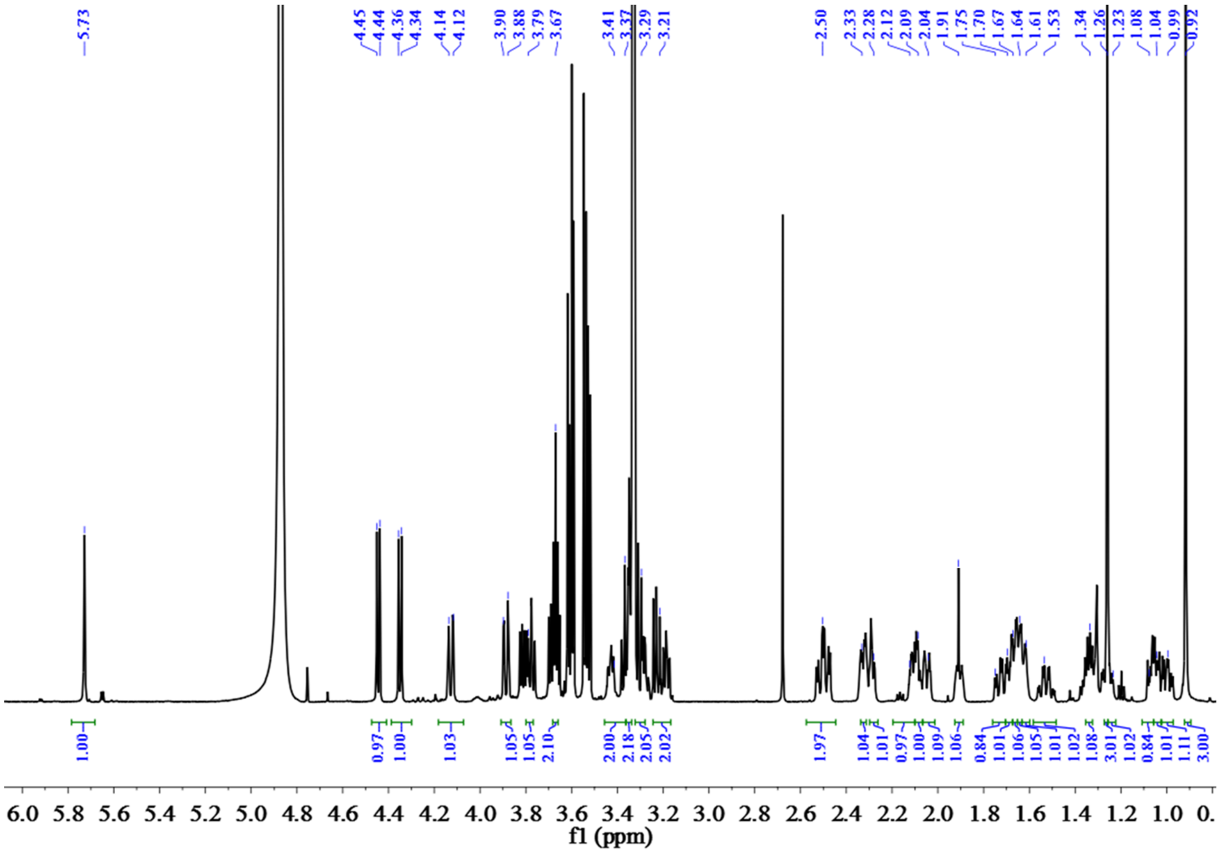

Figure S9. ^{1}H NMR spectrum of **1b** (600 MHz, Methanol-d_4).

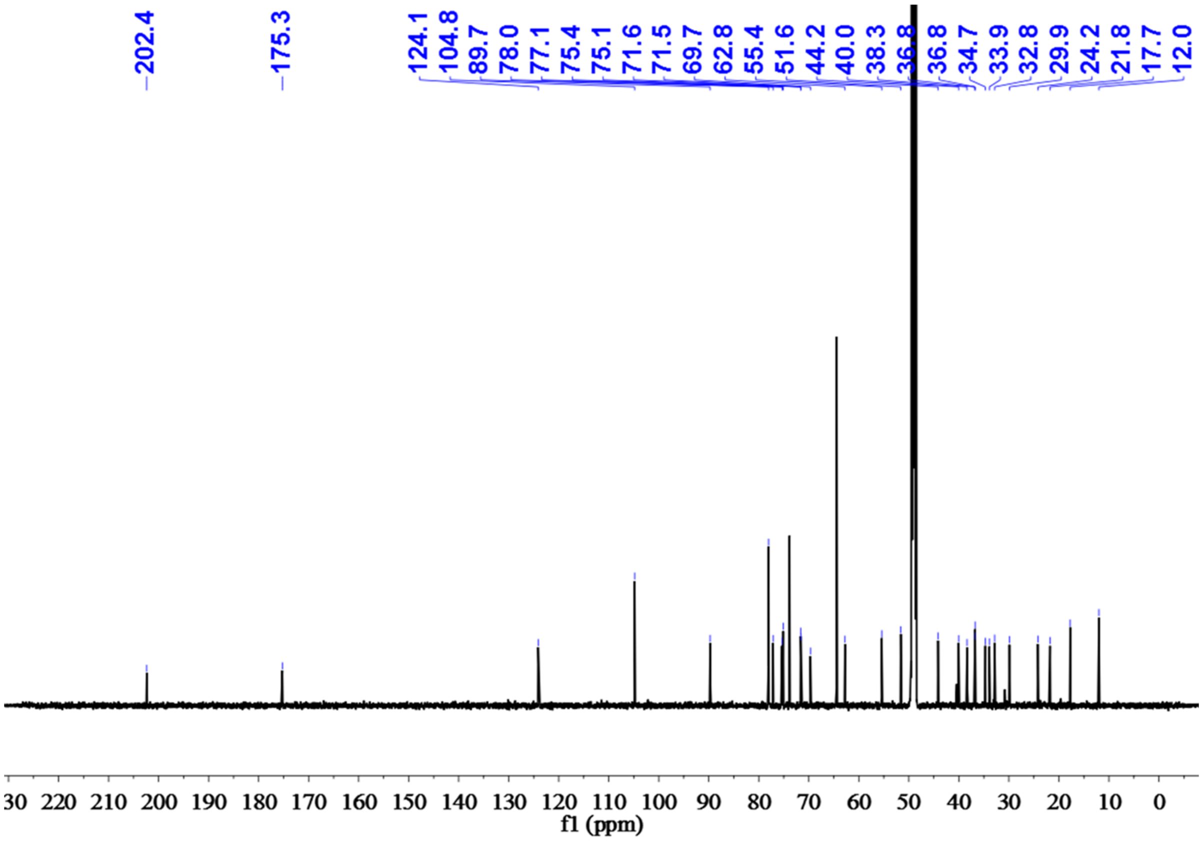

Figure S10. ^{13}C NMR spectrum of **1b** (150 MHz, Methanol-d_4).

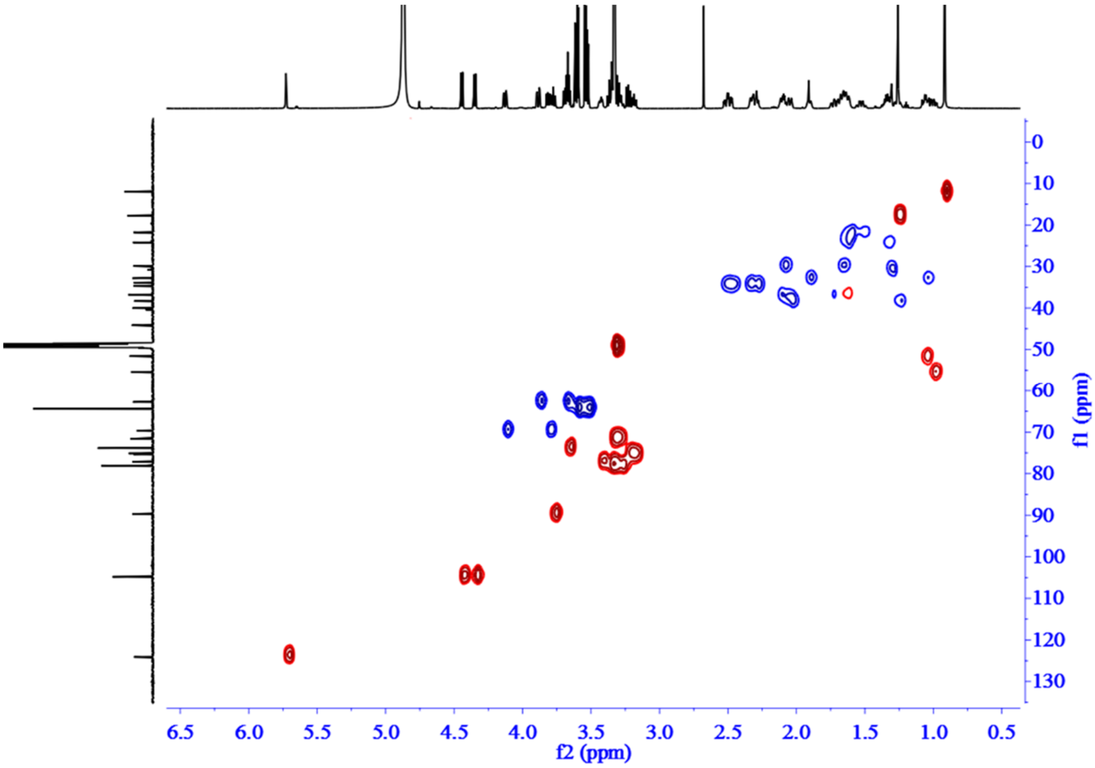

Figure S11. HSQC spectrum of **1b.**

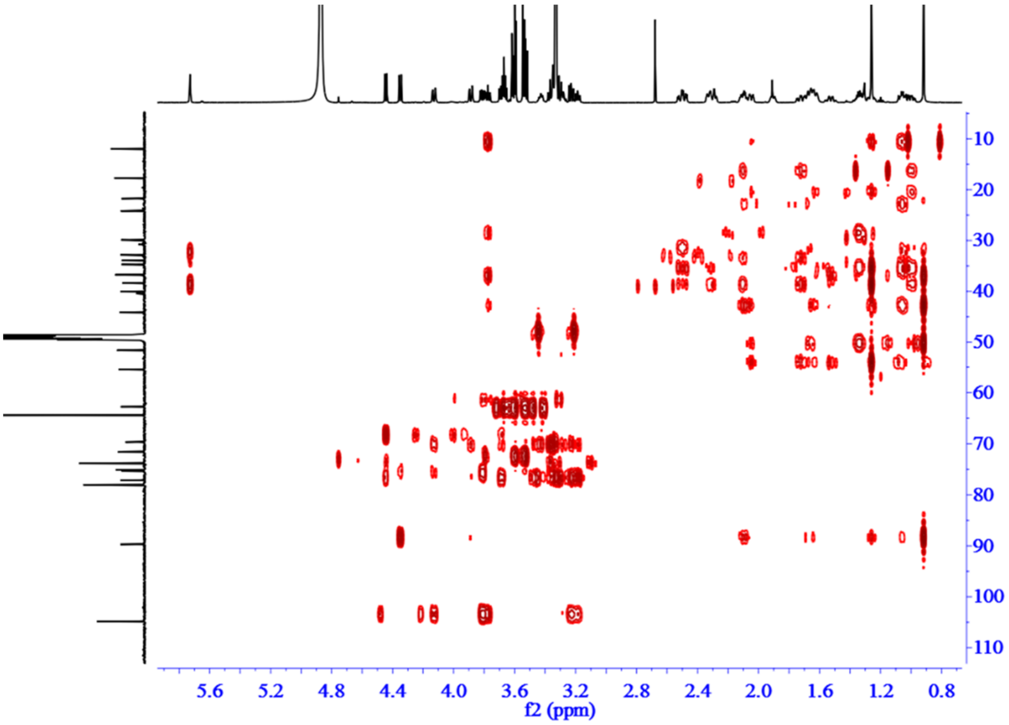

Figure S12. HMBC spectrum of **1b.**

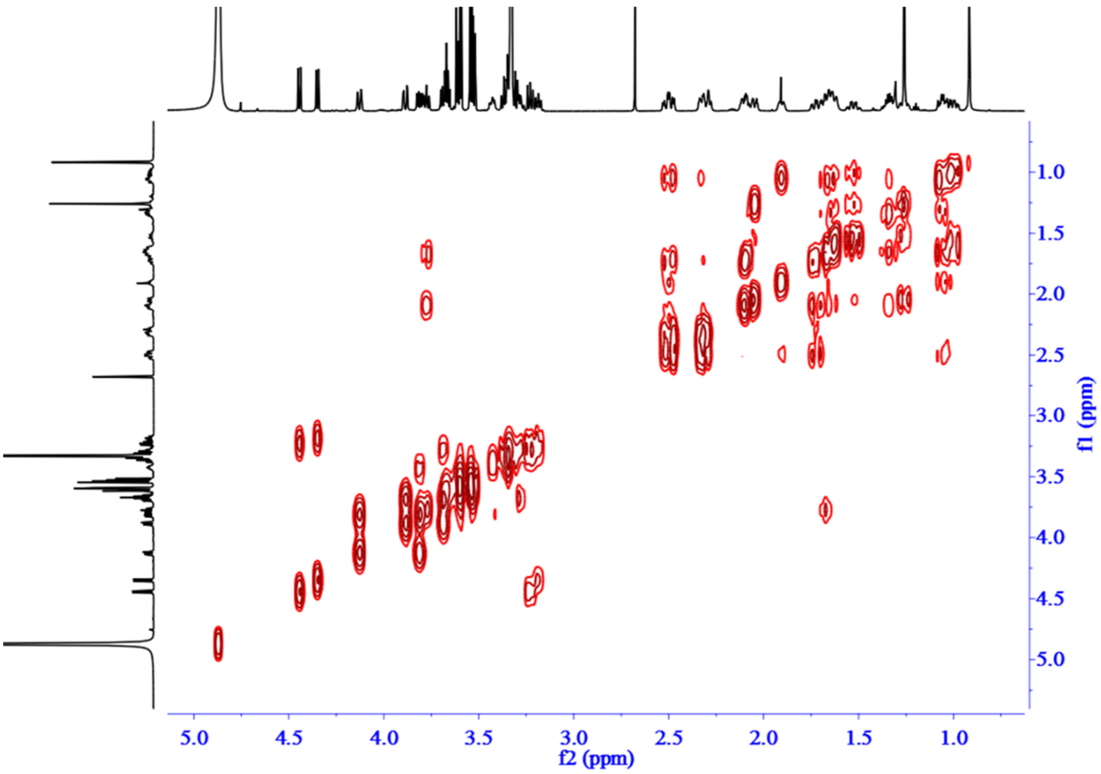

Figure S13. COSY spectrum of **1b.**

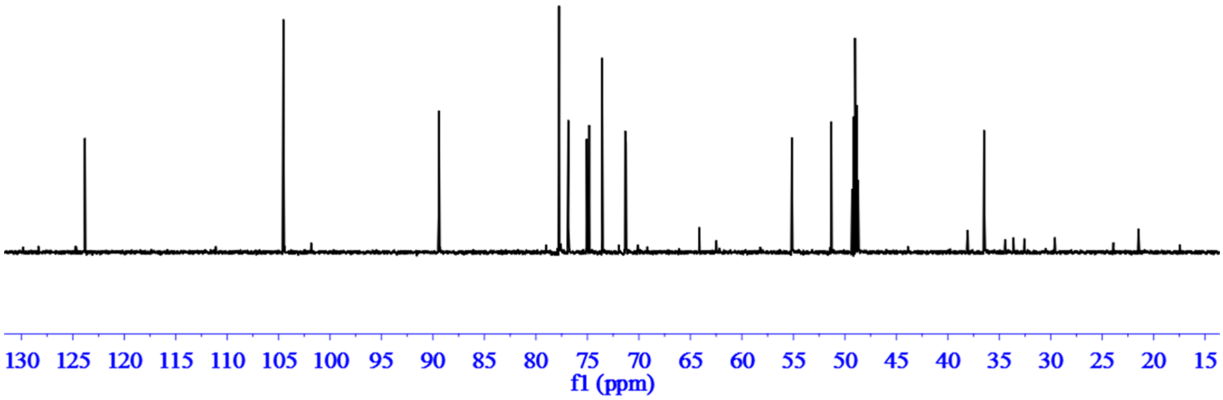

Figure S14. The DEPT-90 ^{13}C spectrum of **1b.**

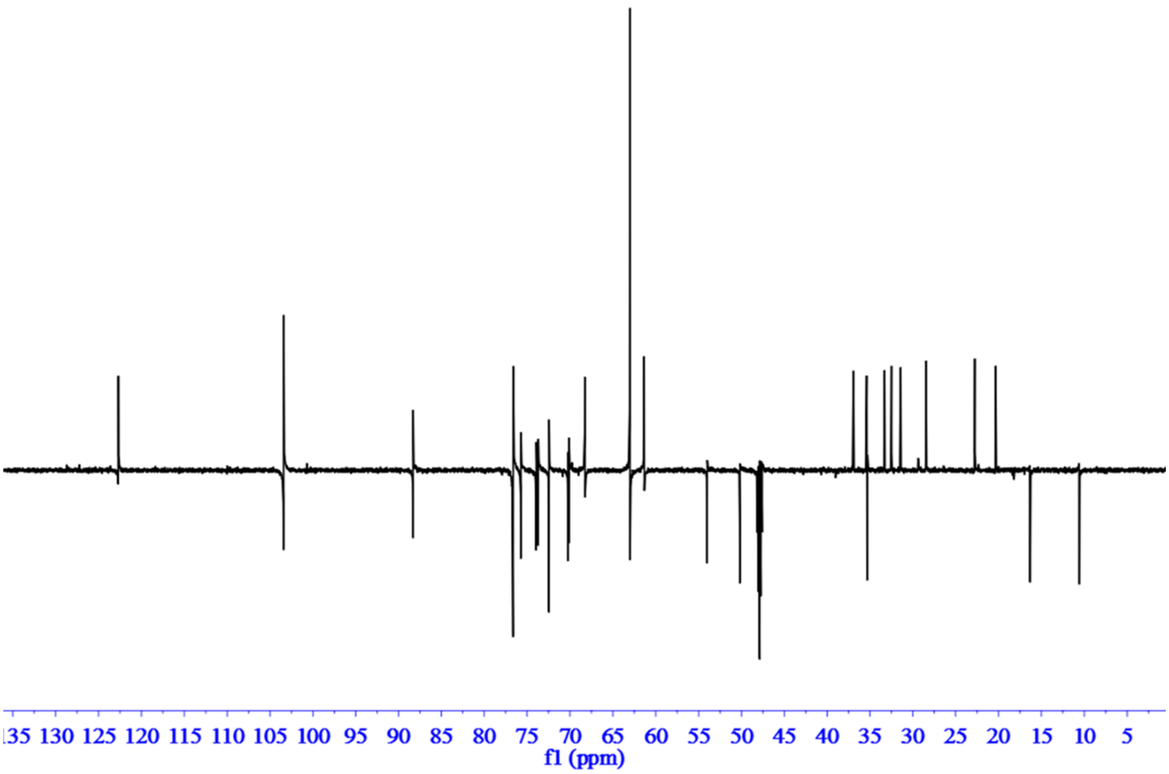

Figure S15. The DEPT-135 ^{13}C spectrum of **1b.**
